# Modeling Community Health with Areal Data: Bayesian Inference with Survey Standard Errors and Spatial Structure

**DOI:** 10.3390/ijerph18136856

**Published:** 2021-06-26

**Authors:** Connor Donegan, Yongwan Chun, Daniel A. Griffith

**Affiliations:** 1 Geospatial Information Sciences, the University of Texas at Dallas, 800 W. Campbell Rd., Richardson, TX 75080-3021, USA; ywchun@utdallas.edu (Y.C.); dagriffith@utdallas.edu (D.A.G.); 2 Population and Data Sciences, University of Texas Southwestern Medical Center, 5323 Harry Hines Blvd, Dallas, TX 75390-9169, USA

**Keywords:** spatial epidemiology, health disparities, Bayesian inference, mortality rates, measurement error, spatial autocorrelation

## Abstract

Epidemiologists and health geographers routinely use small-area survey estimates as covariates to model areal and even individual health outcomes. American Community Survey (ACS) estimates are accompanied by standard errors (SEs), but it is not yet standard practice to use them for evaluating or modeling data reliability. ACS SEs vary systematically across regions, neighborhoods, socioeconomic characteristics, and variables. Failure to consider probable observational error may have substantial impact on the large bodies of literature relying on small-area estimates, including inferential biases and over-confidence in results. The issue is particularly salient for predictive models employed to prioritize communities for service provision or funding allocation. Leveraging the tenets of plausible reasoning and Bayes’ theorem, we propose a conceptual framework and workflow for spatial data analysis with areal survey data, including visual diagnostics and model specifications. To illustrate, we follow Krieger et al.’s (2018) call to routinely use the Index of Concentration at the Extremes (ICE) to monitor spatial inequalities in health and mortality. We construct and examine SEs for the ICE, use visual diagnostics to evaluate our observational error model for the ICE, and then estimate an ICE–mortality gradient by incorporating the latter model into our model of sex-specific, midlife (ages 55–64), all-cause United States county mortality rates. We urge researchers to consider data quality as a criterion for variable selection prior to modeling, and to incorporate data reliability information into their models whenever possible.

## 1. Introduction

Community survey data has long served as an important source of evidence in epidemiology. Du Bois’s, *The Philadelphia Negro* [1,2], among the first modern social epidemiological studies [3], combined original household survey data with United States (U.S.) Census data and municipal health reports to evaluate the role of social conditions, rather than purported biological traits, as determinant of differences in mortality rates among Black and White Philadelphians. Similarly, census tract-level indicators of social class have long substituted for individual-level data in analyses of health inequality [4,5]. Today, epidemiologists and health geographers routinely employ community-level survey data in models of health outcomes because they have access to a variety of geographic data products, including neighborhood-level information. Unfortunately, excitement over access to ‘fine-grained’ geospatial data has generally not been tempered by a realistic assessment of the tradeoffs between data granularity and data quality. For commonly used survey data products such as the American Community Survey (ACS), one should expect data quality to deteriorate as one moves toward smaller spatial scales and toward more detailed concepts and demographic breakdowns. Likewise, one would expect data reliability to vary across demographics and places as a function of social integration/marginalization because standard errors (SEs) are largely a result of sample size and survey response rates. In other words, data quality may often be correlated with the very community characteristics that are of primary interest to investigators. Similar concerns hold for (non-survey based) raster data products, such as *Earth Institute*’s high-spatial-resolution Gridded Population of the World products, notably their sex-specific five-year age group population estimates [6]. This challenge is distinct from, and compounds with, the challenge of inferring underlying patterns of risk from limited observation, such as occurs when big data is disaggregated into small geographic areas and/or multiple demographic groups (see [7]).

This paper proposes a conceptual framework and workflow to support population health research with areal survey data, including visual diagnostics and model specifications. Section 2 provides background information on ACS methodology, including the Census Bureau’s systematic spatial sampling design. We examine a purposeful sample of variables to illustrate how SEs accompanying estimates vary systematically across regions and neighborhoods, by demographic characteristics, and by survey topic (cf. [8,9]). We then review the impacts that sampling error from ACS products may have on descriptive statistics and on inferences when survey estimates are used as covariates [10]. We extend established results on measurement error to a spatial context, arguing that observational error with spatial data has the additional impact of tending to conceal spatial autocorrelation (SA), and, by implication, inflating effective sample size [11,12]. Modeling spatial data with observational error may be a more perilous endeavor than existing research on measurement error alone would imply. Section 3 draws on the findings of the previous sections, as well as previous research on hierarchical Bayesian models (HBMs) for spatial data [13,14,15,16,17,18,19,20], to develop a methodology suitable for modeling community survey data (cf. [19,21]). Appendix A serves as an introduction to plausible reasoning with HBMs, detailing a framework for conceptualizing and building inferential models that incorporate observational uncertainty.

We demonstrate the proposed workflow while building on Krieger, Kim, Feldman, and Waterman’s [22] call to routinely use the Index of Concentration at the Extremes (ICE) [23] to monitor spatial inequalities in health and mortality. We evaluate the reliability of ICE and population at risk data, summarize prior findings on county-level social class–mortality gradients, and then model U.S. county-level, all-cause, sex- and age-specific (55–64 years) mortality rates, comparing results from a “naive” model with our proposed HBM methodology. We urge researchers to routinely incorporate SA and data reliability information into their research workflow, from study design to model criticism and reporting.

## 2. The American Community Survey

The ACS, including the Census Bureau’s subsidiary Puerto Rico Community Survey, is the largest and surely the most widely used source of small-area survey data in the U.S. In accordance with sound scientific practice, the Census Bureau reports estimates together with their SEs whenever possible. These SEs remain woefully underappreciated by the scientific community, in part because of their recent appearance. This section reviews the ACS methodology, examines systematic patterns in the SEs of select ACS variables (extending [8,9]), and discusses some of the implications for models that include ACS estimates as covariates.

### 2.1. A Systematic Spatial Sampling Design

ACS is a continually operating survey of U.S. households that began operations in 2005, and in 2011 increased its target annual sample size from 2.9 to 3.54 million households and increased in-person follow-up rates for non-responding households to 100% in select low-population and primarily Indigenous communities [24] (Ch. 4). The sampling design is systematic by block group (see [25], pp. 23–43, on spatial sampling), and blocks with lower estimated population and lower expected response rates are sampled at higher rates to protect the quality of estimates [24] (Ch. 4). Published estimates are the product of a multi-stage weighting and (for missing, highly implausible, and inconsistent responses) imputation process, and are harmonized with the Census Bureau’s population estimates by sex, age, race, Hispanic origin, and total household units. The three- and five-year estimates are the sum of all weighted responses from the preceding *n* years of surveying [24] (Ch. 11). ACS estimates are accompanied by margins of error (90% confidence intervals) that “reflect the variation in the estimates over all possible samples that could have been selected from the population using the same sampling methodology” not inclusive of possible recording errors and explicitly excluding possible biases in the sampling design [24] (Ch. 12). The Census Bureau calculates margins of error for ACS estimates using the Successive Differences Replication (SDR) method [26,27,28]. This methodology involves repeated sampling from sub-sets of the weighted observations, and then calculating the mean squared error of the replicate estimates from the observed estimate. These procedures can be applied to any function of the estimates such as the ratio, sum, or difference of any two variables. The Census Bureau provides variance replicate tables for a limited number of variables, so that users can calculate SEs for composite variables of their own construction (e.g., deprivation indices) [29].

### 2.2. ACS Standard Errors

This section identifies prominent patterns in SEs for select ACS variables—percent over age 24 with a bachelors degree or higher, median household (HH) income, and percent with health insurance—at the U.S. county level (n=3142) and at the census-tract level for a single county, Milwaukee, Wisconsin (n=296). These are ACS 2018 5-year estimates. The first two of these variables were selected because they are commonly used as indicators of social class, and the third variable was chosen for its particular relevance to public health research. Sampling variability is a function of both sample size (and thus, by extension, survey response rates) and population heterogeneity [8]. Neither response rates nor area heterogeneity are independent of sociopolitical factors, such as social marginalization and patterns of spatial segregation. The findings presented here establish the following characteristics within our sample of variables:

1. SEs contain systematic spatial patterns;
2. Data reliability is not constant across variables;
3. Data describing affluent areas is often of higher reliability than that of impoverished areas; and,
4. Data describing areas estimated to be majority Black, Hispanic, or American Indian tends to be lower quality than that of majority White areas.

**Figure 1:**
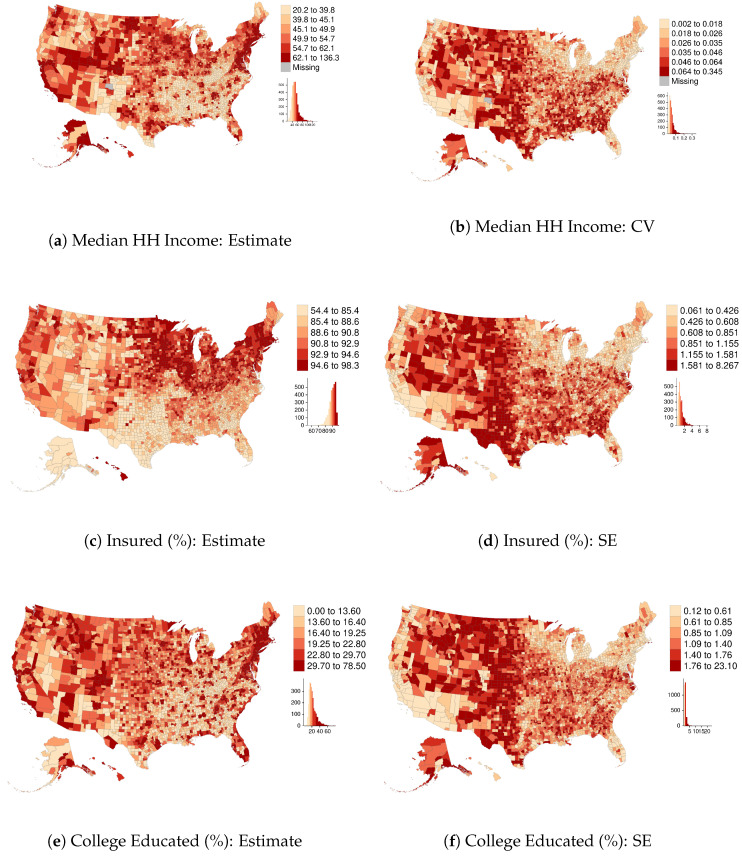
County-level five-year 206 ACS estimates and their data quality measures (CV for income, SEs for the others) for select variables. Median HH income is reported in thousands of dollars.

**Figure 2:**
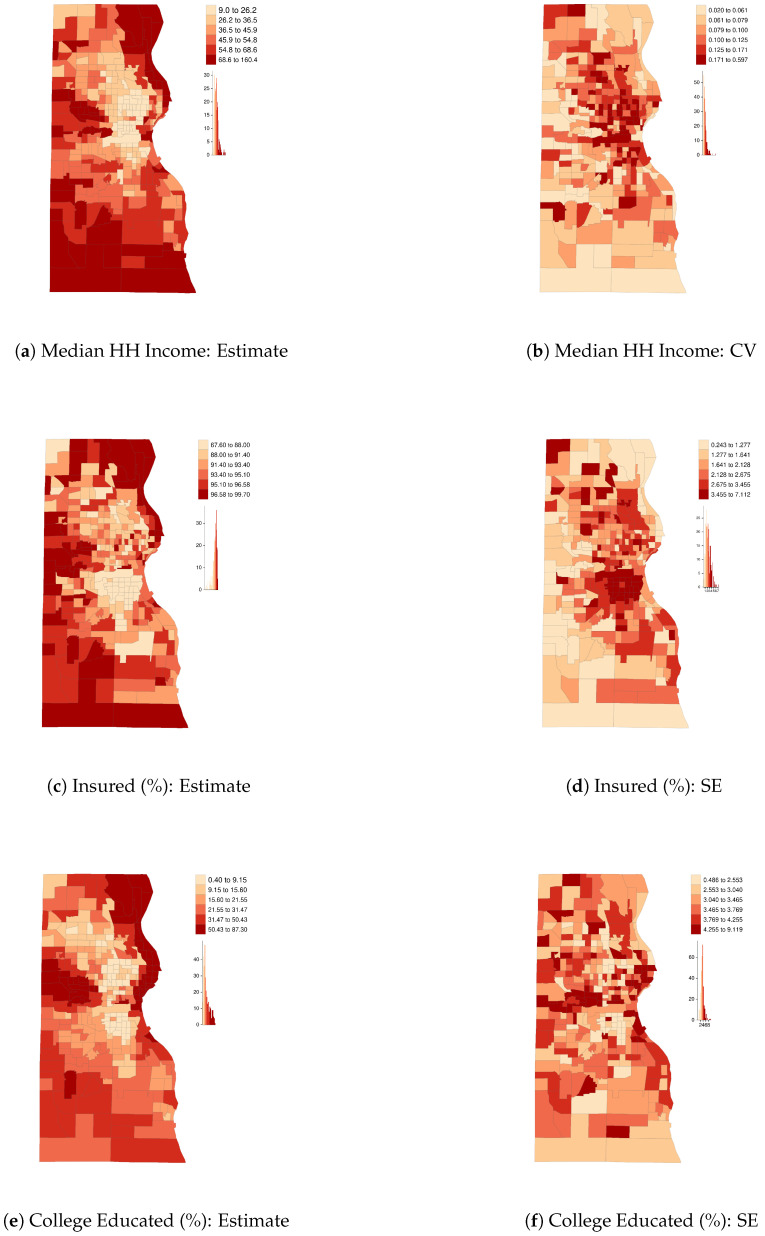
Milwaukee County, Wisconsin census tract-level five-year 2018 ACS estimates and their data quality measure (CV for income, SEs for the others) for select variables. Median HH income is reported in thousands of dollars.

**Figure 3:**
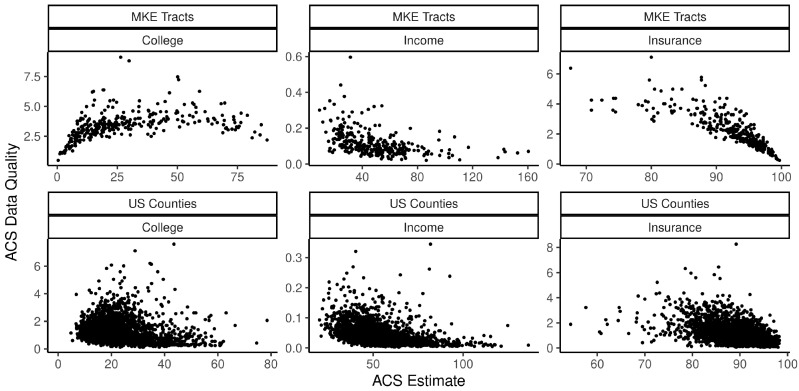
ACS estimates and their data quality measure (CV for income, SEs for the others). Median HH income is reported in thousands of dollars. A single outlying observation of college education at the county level is excluded from the plot (Loving County, Texas, estimated 0% college educated, SE = 23.1).

**Figure 4:**
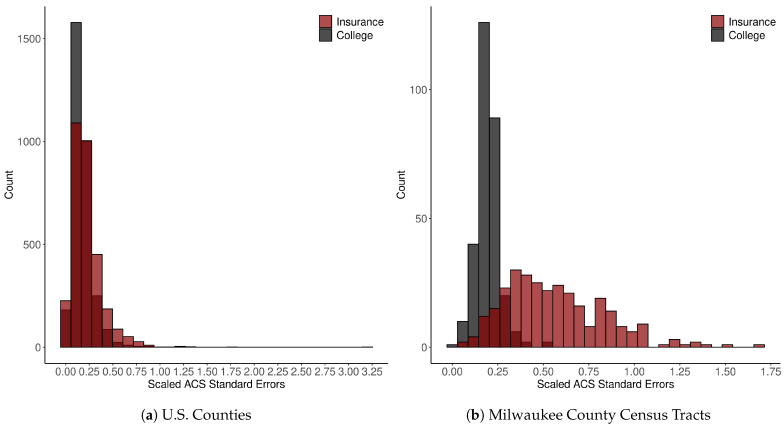
Histograms of scaled ACS SEs for percent insured and percent college educated. The values on the horizontal represent the ratio of each respective SE to the MAD of the variable of interest across its respective geographic domain (U.S. counties or Milwaukee County census tracts).

Points 1 and 3 are strikingly clear from the maps of ACS data quality in Figure 1 and Figure 2. For example, counties in the northeastern region are estimated to have high median HH incomes and low coefficients of variation ($\mathrm{CV}=\frac{\mathrm{SE}}{\mathrm{Estimate}}$), whereas Appalachia and the Deep South have low estimated incomes and relatively high CVs. Analogous spatial patterns appear across impoverished and affluent areas of Milwaukee County. These observations are consistent with previous findings [8,9], including, for example, that ACS estimates of median Black HH income “are especially bad for the poorest 15 percent of census tracts” [8] (p. 152). Table 1 quantifies the degree of SA in each variable and in their respective measures of reliability (SE or CV) using the SA parameter from an intercept-only simultaneous autoregressive (SAR) model. The auto-normal SAR specification models *n* observations of an outcome, *y*, using the multivariate normal distribution as the likelihood function, instead of specifying the likelihood as the product of *n* independent, univariate normal distributions. The covariance matrix, Σ, incorporates a row-standardized spatial connectivity matrix, W, with parameter ρ accounting for the nature and degree of SA, and σ for the usual scale parameter: $\Sigma =\sigma^{2}{\left[\left(I-\rho W^{\prime }\right)\left(I-\rho W\right)\right]}^{-1}$ [20] (pp. 198–200). The SEs of all three variables at both scales show moderately high levels of SA (from 0.46 to 0.69), similar to many other socioeconomic and demographic variables. Table 2 reports relative data quality (as measured by their median value) by majority racial-ethnic group, with majority White areas serving as the reference category; Figure 3 plots data quality against estimates themselves. We see that all three variables tend to be of higher quality in majority White areas and affluent areas, with the exception of educational attainment in Milwaukee, for which the inverse holds. This is almost certainly related to the degree of homogeneity of the population—standard errors are smallest in census tracts where the proportion of college graduates is nearest to zero. Similarly, the SEs for health insurance coverage shrink as the estimates approach one. This is consistent with the formula for the sampling variance of a proportion, *p*, from the binomial distribution, VAR(p)=p∗(1−p)/n, where *n* is the sample size. Figure 4 shows that data quality can be highly variable across survey topics—estimates of tract-level insurance coverage are of substantially inferior quality than those of educational attainment. Notice, many of the SEs are greater than the median absolute deviation (MAD) of the variable itself. These findings indicate that data quality is itself a social variable.

### 2.3. Implications

The inter-related, systematic patterns present in ACS data quality may impact inferences in complex ways. Past findings regarding measurement error provide a useful starting point, if only to indicate the minimal severity of the problem. Sampling error is most appropriately modeled following the framework of “classical” measurement error, which represents observations, X, as the sum of the actual values, X, and errors, Δ,

(1) X=X+Δ

where each is an n×k matrix, where *n* is the number of areas observed and *k* is the number of variables. Three important and well-known results follow from this representation [10] (pp. 1–64):

1. Additive error tends to increase sample variance, leading to exaggerated confidence in regression estimates;
2. the additional variability *tends to* attenuate bivariate correlations and mask non-linear relations; and,
3. in multivariate models, observational error may result in a change of sign, attenuation, or exaggeration of coefficient estimates at any sample size.

**Figure 5:**
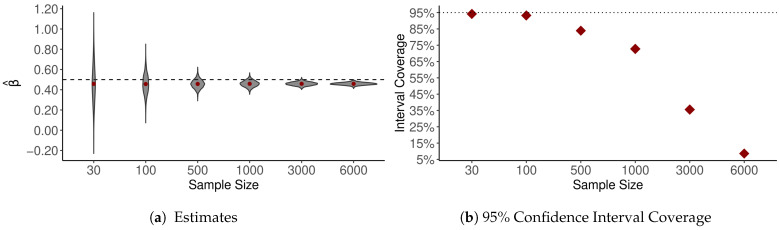
Bivariate regression coefficient estimates from simulated data with additive measurement error. For each of *M* = 5000 iterations, *n* = {30, 100, 500, 1000, 3000, 6000} values of *x* were drawn, *x* ~ *N*(0,1); *y_i_* was calculated as α + *x_i_* ∗ β + *e_i_* where *e_i_* ~ *N*(0,1) and β = 0.5; then y was regressed on z, where *z_i_* = *x_i_* + *u_i_*, *u_i_* ~ *N*(0,0.3).

Figure 5 illustrates what is meant by a *tendency* toward attenuation in bivariate regression—observed relations may still be attenuated or exaggerated, but with large numbers of observations, attenuation becomes a near certainty and confidence intervals become wildly misleading (cf. [30,31]). With spatial data, such as small-area ACS estimates, two additional insights follow directly from Equation (1):

4. Observational error tends to decrease the degree of observed SA; and,
5. Spatial variation in data quality tends to produce spatial variation in the analytical consequences of observational error.

The latter observation has implications for models that attempt to infer spatially varying relations among variables, such as geographically weighted regression [32]. Geographic variation in the corruption of observations may produce spurious geographic variation in relations between variables (geographically varying bias). The former observation indicates that our measures of SA may often be underestimated, which implies an over-estimation of effective sample size or, to rephrase, an improper weighting of evidence.

## 3. Spatial HBMs for Survey Data

This section presents our proposed methodology for modeling small-area survey data. We illustrate and validate the analytical argument by comparing rival models for a single variable, health insurance coverage, and we provide a set of visual diagnostic plots to evaluate our model. We then use our methodology to compare raw ACS estimates for select variables, X, with the probability distributions for their respective true values, X, noting inferential problems that may be introduced by the confluence of SA and observational error.

### 3.1. Prior Information and Model Specification

HBMs are built by successive application of the product rule for expressing the joint probability of multiple propositions (see Appendix A for additional details). Bayes’ theorem provides a method for calculating the probability of a proposition given data, *X*, and any relevant (prior) information, *I*. The probability of the proposition, after seeing the data, is known as the ‘posterior probability’, leading to the summary expression of Bayes’ theorem,

(2) Posterior∝Likelihood×Prior.

When considering observational error, or complex spatial or spatio-temporal information, the joint probability expands into numerous terms, leaving Equation (2) wanting for clarity. Thus Clayton [13,14,15,16] proposed to factor epidemiological HBMs into,

(3) Posterior∝[Measurementmodel]×[Diseasemodel]×[Exposuremodel],

whereas Berliner [17,18,20] introduced the generic terms,

(4) Posterior∝[Datamodel]×[Processmodel]×[Parametermodel].

In both cases, the concept is identical: the first term incorporates information about the measurement or observational process that may have introduced a difference between the state of reality, X, and our record of it, X. The process model encodes substantive knowledge of the process under study, and the parameter model encodes contextual knowledge about the possible states of that process.

Building HBMs for ACS data requires specification of three probability models: the data/measurement model, p(X|X,I), the process model, p(X|ζ,I) where ζ are process parameters, and the parameter/exposure model, p(ζ|I). For the first, we assign to the errors, Δ, a Gaussian probability density with variance, $\sigma^{2}$, equal to the square of the SEs of the estimates, $S^{2}$, such that X∼Gauss(X,S). This specification may be justified for continuous variables by the maximum entropy principle insofar as our information for each observation consists of only location (estimate) and scale (SE) parameters [33,34]. The Census Bureau’s practice of calculating 90% margins of error by multiplying the SEs by 1.645 also implies a Gaussian probability distribution for the sampling errors. For the process model, p(X|ζ,I), we require a probability model that incorporates the most pertinent and generalizable information we have about socioeconomic and health variables. Foremost are the following social and economic characteristics of contemporary society:

1. Polarization, such that relatively extreme values are not unexpected; and,
2. Segregation, such that most social and economic variables display moderate to strong SA.

The first postulate conflicts with the Gaussian model, which places very low probability on outliers and extreme values. The second observation eliminates the uniform distribution from consideration, because it would prevent us from incorporating SA. The uniform distribution would also result in models that place high probability on values that are implausibly far from the range of observed estimates, given that ACS SEs can be quite large (polarization does not imply unrestricted variation).

An auto-Gaussian model that incorporates SA in the covariance matrix automatically increases the probability of tail-area (extreme) values relative to an independent Gaussian model, while also placing low probability on outliers relative to the local area mean. This model incorporates both postulates simultaneously because outliers (relative to the global mean) tend to cluster together at commonly employed units of aggregation (census tracts, counties, and states). Similar to Kang, Liu, and Cressie [19], we suggest the following model specification for small-area survey data:

(5) $$\begin{array}{cc} {}[\mathrm{Data}\;\mathrm{model}]:\;X & ∼Gauss(X,S)\\ {}[\mathrm{Process}\;\mathrm{model}]:\;X & ∼MVGauss(1\mu ,{\left(I-\rho W\right)}^{-1}M)\\ {}[\mathrm{Parameter}\;\mathrm{model}]:\;\mu & ∼Gauss(*,*)\\ \tau & ∼Student^{+}\left(*,*,*\right)\\ \rho & ∼Uniform(\frac{1}{\lambda_{min}},\frac{1}{\lambda_{max}}). \end{array}$$

The process model for X is an auto-Gaussian model with a conditional autoregressive (CAR) specification of the covariance matrix [20] (pp. 167–203) where μ is a constant mean multiplied by an n×1 vector of ones, $M=\tau^{2}D^{-1}$ is a diagonal matrix of conditional variances consisting of the inverse of the number of neighbors of each respective areal unit $D_{i,i}^{-1}$ times a scale parameter, $\tau^{2}$, ρ is a parameter accounting for the nature and degree of SA, and W is a row-standardized connectivity matrix with zeroes on the diagonal (also given as data). W is specified such that any element $W_{i,j}$ equals $D_{i,i}^{-1}$ if the ith and jth observations are neighbors, and zero otherwise (This is just one valid specification of the CAR model. For others, see [20,35]). We define neighbors using the queen contiguity condition [35] (p. 89), [36]. The range of permissible values for the SA parameter ρ is determined by the smallest and largest eigenvalues ($\lambda_{min}^{-1}$, $\lambda_{max}^{-1}$) of the matrix $M^{-1/2}WM^{1/2}$ [20]. The * symbol indicates prior parameters to be specified relative to the problem at hand. Finally, the models must reflect any natural boundaries in the data (e.g., percentages range only from zero to one hundred). Such truncated distributions are easily programmed into MCMC algorithms by placing boundaries on the parameter space. We experimented with one alternative specification: a Student’s *t* model with spatially varying mean, using eigenvector spatial filtering [37,38]. We conclude that the auto-Gaussian model performs similarly to the spatial *t* model, but with substantial computational advantages—it is fairly efficient with moderately large *n* (n≈3000) using Stan [39].

### 3.2. Model Evaluation

Here we model percent of residents in Milwaukee County census tracts who have health insurance (ACS variable DP03_0096P, see Figure 2c,d). As noted, this variable has large SEs, which renders results particularly sensitive to model specification error. We leverage this fact to highlight the differences between models. We compare results from the auto-Gaussian specification of Equation (5) with a non-spatial Gaussian model. For each model, we examine the differences, $\hat{\Delta }$, between the mean of their respective posterior distributions, p(X|X,S,I), and their raw ACS estimates. In other words, ${\hat{\Delta }}_{i}$ is the mean of the posterior distribution of the error, $\Delta_{i}=X_{i}-X_{i}$. Diagnostic plots reveal that the non-spatial model produces systematically biased inferential patterns, but results from the auto-Gaussian model appear reasonable.

**Figure 6:**
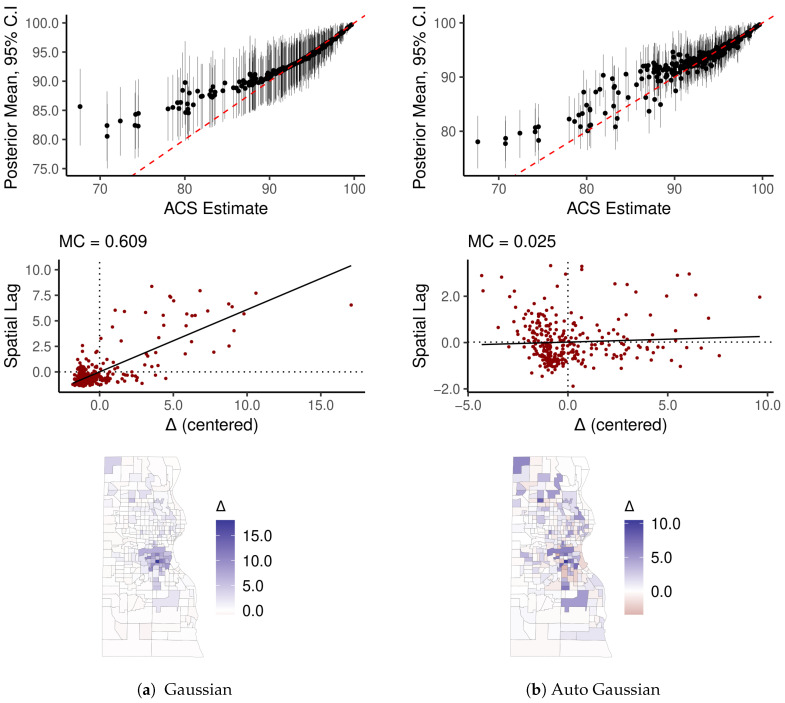
Diagnostics for observational error models of percent insured by Milwaukee County census tract. Δ is the difference between posterior means and their respective raw ACS estimates.

Each panel in Figure 6 contains three diagnostic plots. The top figure is a point-interval plot of the raw ACS estimates against a summary of their respective posterior distributions (mean and 95% credible intervals (CI)), highlighting that the non-spatial model imposes unidirectional shrinkage toward the global mean value on the estimates. Below the point-interval plot is a Moran scatter plot (see [25,35,40]), which reveals that the non-spatial model has moderately strong SA in its $\hat{\Delta }$ values. The map of $\hat{\Delta }$ reveals that a distinct sociospatial pattern underlies the SA—the majority Black and Hispanic inner city tracts all have ${\hat{\Delta }}_{i}>0$. The auto-Gaussian model, by contrast, pulls estimates with large SEs toward the local mean, and, as a result, $\hat{\Delta }$ reveals no conspicuous or concerning spatial pattern. Because the spatial model incorporates additional information relative to the non-spatial model (and the degree of SA in these particular variables is strong), the posterior distributions tend to be narrower, reflecting a greater degree of confidence in results.

### 3.3. Examining Implications

Here we report differences in the mean, dispersion, and SA between the raw ACS point estimates and their posterior distributions obtained employing the proposed CAR model. Our concern is that using raw ACS variables as covariates is leading researchers to become overly confident in their model results, due to a combination of inflated sample variance and deflated SA.

We utilize ACS data on three variables for Milwaukee County census tracts to fit the model, and we report summary statistics in Table 3. Median HH income was log-transformed to better suit our model, and its SEs were appropriately transformed as well. (SEs for $log(x_{i})$ may be approximated by the transformation $s_{x_{i}}\to \frac{1}{x_{i}}s_{x_{i}}$. We applied a simple Monte Carlo method to construct SEs for the transformed variate.) For each variable, the mean, standard deviation, and degree of SA ρ (obtained from a SAR model) were calculated for each sample from the joint posterior distribution of parameters. For example, the posterior distribution for the standard deviation of tract-level, log-transformed median HH income has a mean of 0.46, with a 95% CI of [0.45,0.48], compared to a raw value of 0.49. Only small differences appear between the raw and modeled values of percent college educated. Yet the posterior distribution for insurance coverage has markedly greater SA ($\hat{\rho }=0.89$, 95% CI: [0.86,0.92]) than the raw ACS estimate ($\hat{\rho }=0.82$), and its standard deviation decreased by 20%—from 5.67 to 4.59 (CI: [4.22,4.96]); hence, a variance decline of ≈34%, from 32 to 21.

The amount of meaningful information present in the raw estimates of percent insured was twice inflated—once by sampling error directly, and again by the obfuscation of SA. Consider the concept of effective sample size $n^{*}$, the number of equivalent independent observations required to obtain the same information content as an autocorrelated sample [11] (p. 15). For data well-modeled by the Gaussian distribution, ρ=0.82 converts a nominal sample of n=296 to $n^{*}\approx 23$, whereas a value of ρ=0.9 has $n^{*}\approx 12$ [12] (Equation (3)). Hence, strong SA in covariates causes measures of uncertainty to deteriorate in quality, whether they be p-values or Bayesian CIs, with or without a spatial model describing the outcome variable [37,38,41,42]. Thus, the results presented in this section provide additional motivation to carefully evaluate data quality as a criterion for variable selection, and to properly model both SA and observational error.

## 4. Modeling U.S. County Mid-Life Mortality

In this section, we model all-cause sex-specific U.S. county mortality rates for ages 55–64, and estimate the ICE–mortality gradient following Krieger et al. [22]. We exclude Alaska due to substantial differences in the state’s county equivalents. Approximately half of Alaska’s area is a single county equivalent, larger in area that any other state, which introduces the modifiable areal unit problem, in its worst possible materialization. If our primary purpose in this paper were to model U.S. mortality rates, then we would incorporate Alaska’s county equivalents through, perhaps, its own independent model [43]. Using ACS SEs and our data model, we find that the quality of county-level ICE estimates is neither particularly poor nor negligible. We compare results from our proposed spatial HBM of mortality rates to a naive model that does not consider observational uncertainty but is otherwise identical. We find that the naive model underestimates the ICE–mortality gradient and generally produces more narrow CIs for the county mortality rates. Some counties have sizable differences in predicted mortality rates while in dozens of counties the increase in posterior uncertainty is substantial.

### 4.1. Data and Prior Information

**Figure 7:**
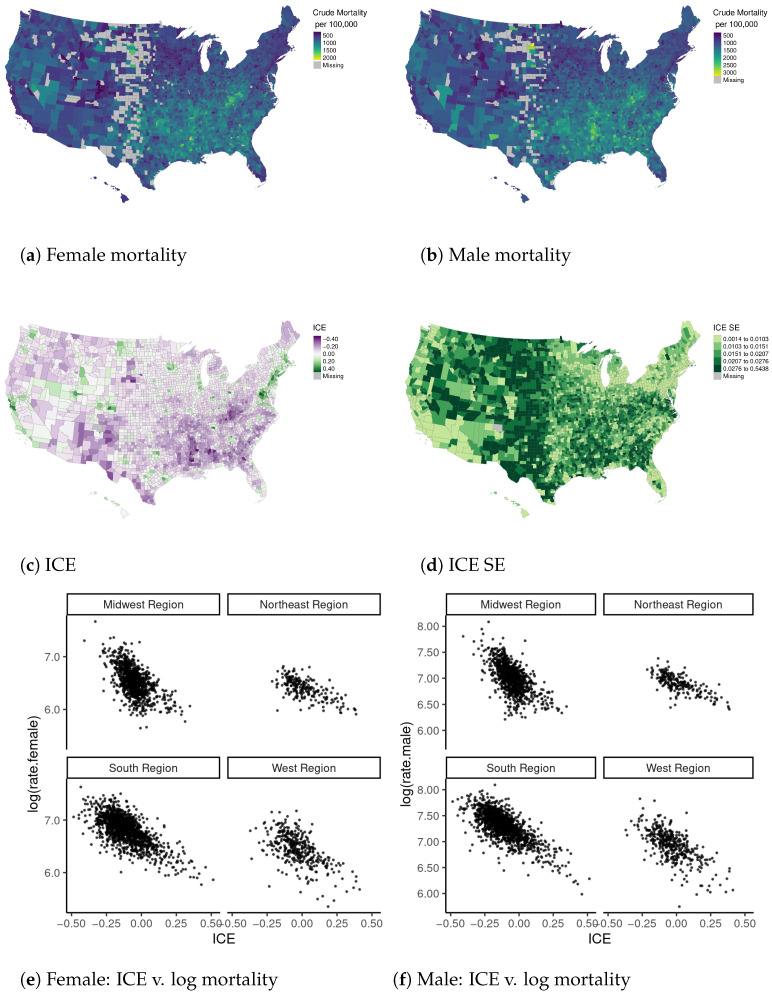
Raw U.S. county all-cause mortality rates by sex for ages 55–64, ICE estimates and their SEs, and scatter plots relating the ICE to the natural logarithm of the mortality rates by Census region.

**Figure 8:**
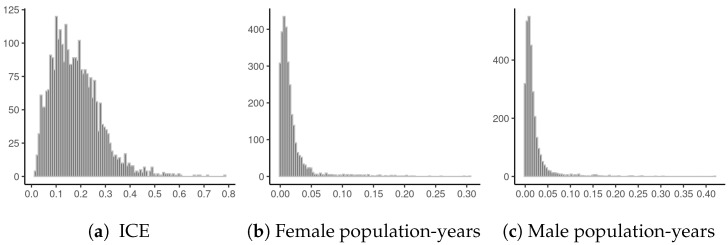
Data quality for county ACS variables. Scaled SEs $\left(\frac{SE(x_{i})}{MAD(x)}\right)$ are shown for the ICE estimates, and CVs are shown for population at risk data.

**Figure 9:**
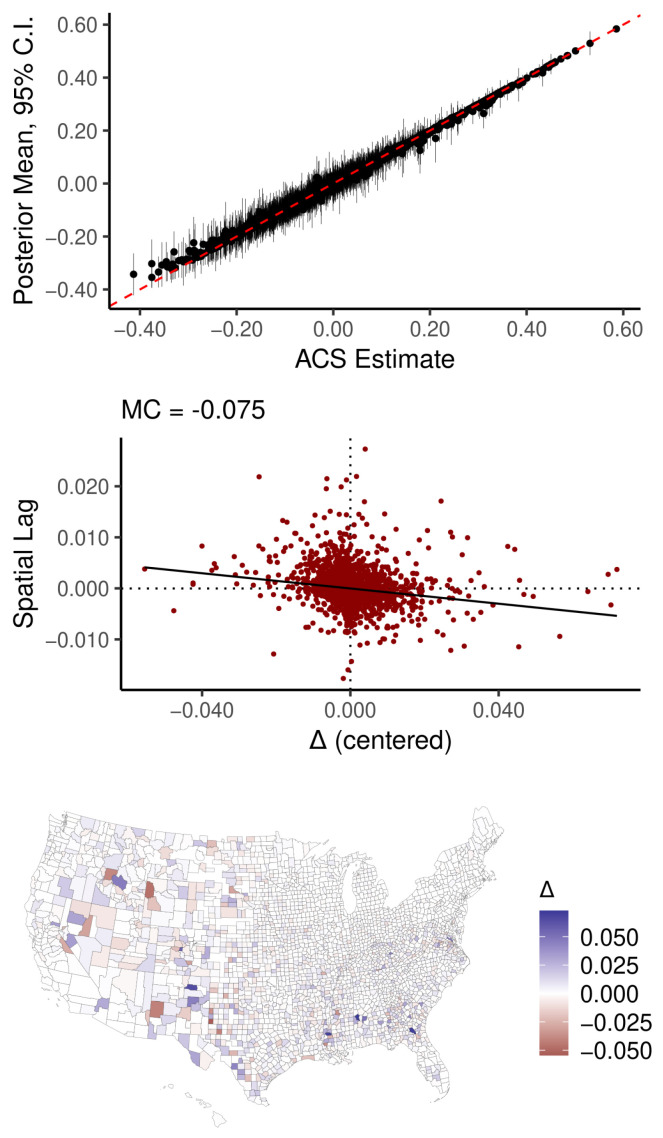
Diagnostics from the auto-Gaussian data model for county-level ICE.

We gathered county-level all-cause mortality and population-at-risk data from CDC Wonder by sex for ages 55–64, aggregating over years 2014 through 2018 (Figure 7a,b) [44]. We dropped counties for which the mortality data is censored, and we also dropped one area that is missing an ICE value. Thus, our analysis includes n=2984 counties with male mortality data, and n=2875 counties with female mortality data. We manually updated the connectivity structure to link together some rural, low-population counties with missing observations between them (see online supplementary material for additional information). We used the Census Bureau’s variance replicate tables [29] to calculate the ICE by county with appropriate SEs (Figure 7c,d). The ICE is calculated as

(6) $$\mathrm{ICE}=\frac{\mathrm{No}.\;\mathrm{highest}\;\mathrm{income}\;\mathrm{households}-\mathrm{No}.\;\mathrm{lowest}\;\mathrm{income}\;\mathrm{households}}{\mathrm{Total}\;\mathrm{no}.\;\mathrm{households}},$$

with threshold incomes for lowest and highest income groups set to $20,000 and $125,000, respectively (following Krieger et al. [22]). The ICE ranges from −1 to 1, with 1 corresponding to a population where all HH incomes are ≥$125,000 and −1 corresponding to a population where all HH incomes are <$20,000. Figure 8a plots the SEs of each ICE estimate divided by the MAD of the ICE itself (excluding counties with censored mortality data). The median SE is 0.17 times the MAD, and 50% of the SEs are between 0.11 and 0.24 times the MAD; the largest SE is 0.78 times the MAD. Figure 9 provides diagnostics for our auto-Gaussian data model for the ICE. The ${\hat{\Delta }}_{i}$ values are not particularly large and have no SA, which is reassuring. Examination of CVs for the estimated population-years at risk shows that the vast majority of CVs are <0.05 (Figure 8b,c).

To gather prior information on the ICE–mortality gradient, we searched PubMed for published research containing “all cause mortality” or “premature mortality” as well as “county” in its title or abstract ((((“all cause mortality” [Title/Abstract])) OR (“premature mortality” [Title/Abstract])) AND (“county” [Title/Abstract])). Of 310 results, 24 appeared potentially relevant and were selected for closer inspection. Eight of these studies reported findings on the degree of inequality in county mortality rates, although no two studies employed the same measurement of inequality (see Table A1). We measure inequality by the relative index of inequality (RII): the mortality rate ratio of the most disadvantaged over the most advantaged group. RII maintains conceptual consistency across rate ratios by keeping the most disadvantaged group in the numerator. Thus the quintile-based RII (${\mathrm{RII}}_{5}$) with counties ordered by mortality rates is $\frac{p80}{p20}$, whereas the comparable quantity for counties ordered by the ICE is $\frac{p20}{p80}$. Two studies comparing the bottom to the top quartile of counties (${\mathrm{RII}}_{4}$) ordered by socioeconomic variables [45,46] found ${\mathrm{RII}}_{4}$ values of 1.22 and 1.41, respectively; those comparing the first to fifth quintiles of counties (${\mathrm{RII}}_{5}$) ordered by socioeconomic variables [47,48,49] found ${\mathrm{RII}}_{5}$ between 1.5 and 1.8; and, those reporting RIIs by decile [50] or other tail-area grouping [51,52] found RIIs between 1.6 and 2.7.

**Figure 10:**
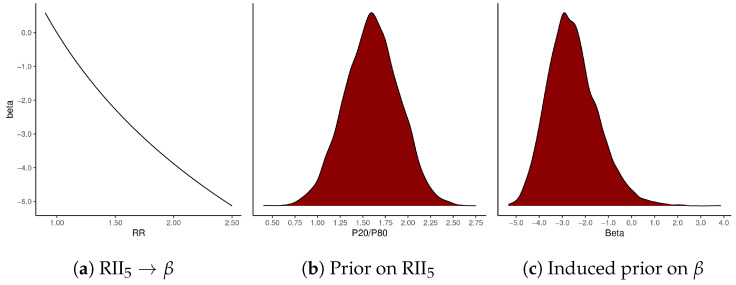
The prior probability model for *β*, the ICE-mortality gradient. RII_5_ is the *p*20/*p*80 mortality rate ratio, with counties ordered by their ICE.

Based on this semi-formal review, we expect ${\mathrm{RII}}_{5}$—comparing the 20th percentile (p20) to the 80th percentile (p80) of counties ordered by the ICE—to be greater than unity, and we would be surprised if it were larger than 2.2 (for further discussion, see Appendix B). Our exploratory analysis of the data, including the scatter plots of the ICE against log-mortality rates by Census region in Figure 7e,f, indicate that log-mortality rates show an approximately linear relationship with the ICE. This result means that any value of ${\mathrm{RII}}_{5}$ can be converted into its corresponding coefficient β from a log-linear model: $\beta =\frac{d\log(y)}{dx}=\frac{\log({\mathrm{RII}}_{5}^{-1})}{p80-p20}$ (see Figure 10a). The value dx=p80−p20 can be calculated from our ICE data model, and equals 0.179[0.176,0.182]. To encode our substantive prior information about ${\mathrm{RII}}_{5}$ into a probability distribution for β, we use the following model:

(7) $$\begin{array}{c} {\mathrm{RII}}_{5}∼Gauss\left(1.6,0.3\right)\\ \beta =\frac{\log({\mathrm{RII}}_{5}^{-1})}{0.179}. \end{array}$$

**Figure 11:**
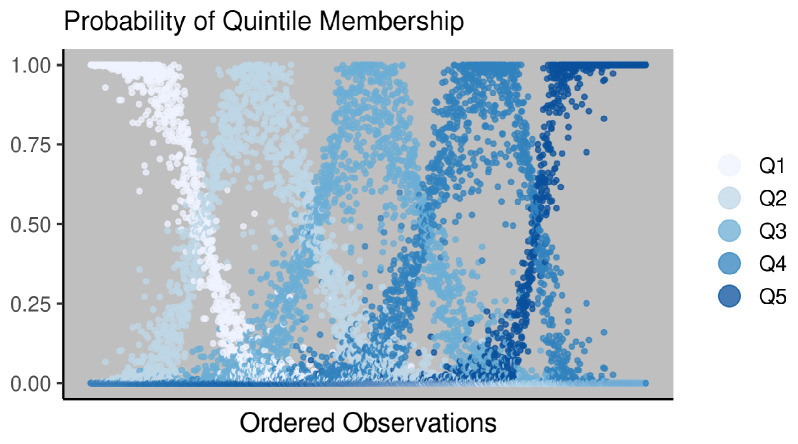
Uncertainty of quintile membership for county ICE observations. Results are derived from the joint probability distribution of the auto-Gaussian ICE data model.

Figure 10b is a density plot of the Gaussian prior on ${\mathrm{RII}}_{5}$, whereas Figure 10c shows how that density transfers to values of β. Most of the probability density is assigned to values of β between −4.5 and −1. We do not convert our continuous measure of the ICE into discrete quintiles or other bins before modeling because that modification would arbitrarily delete data in our possession, and, as Figure 11 illustrates, our observational uncertainty regarding ICE values implies considerable uncertainty regarding to which quintile many observations belong. The number of observations with ambiguous membership in either the first or the fifth quintile, say those having a probability between 0.2 and 0.8 of belonging to either one, is 443 or 15% of our observations on male mortality.

### 4.2. Process and Parameter Models

Because the mortality data, Y, for each respective sex consists of a vector of counts of a rare outcome (relative to the size of the population at risk), we assign a Poisson probability distribution to the likelihood, with mean and variance equal to the parameter μ=λ·P, the elementwise product of rates, λ, and population-years at risk, P. We model male and female mortality independently, applying the same model specification to each. The logs of the male and female mortality rates show moderately strong SA, both with Moran coefficients (MC) of MC=0.56. The ICE (X) also has strong SA, with MC=0.643[0.638,0.648]. We model SA in the outcome using the Besag-York-Mollié (BYM) specification [53,54]. Whereas the CAR model from Equation (5) combines spatial trends (ρW) and independent variation (M) into a single covariance matrix, the BYM model achieves computational efficiency by splitting these components of the model into two separate parameter models. This separation requires two parameter vectors: an SA term, ϕ, plus the non-spatial term, θ. The intrinsic CAR (ICAR) prior is placed on ϕ, where the SA parameter ρ is implicitly fixed to 1; hence it places high prior probability on smooth variation. Setting ρ=1 also renders the joint probability distribution of ϕ improper, in the sense that it does not integrate to one. This is addressed by constraining the values of ϕ to sum to zero (see [35], pp. 246–247). We implement this constraint following Morris et al.’s [54,55] method.

The BYM model captures additional variation around the spatial trend by assigning a Gaussian prior with unknown scale to θ. The relative influence of the two terms is controlled by their respective scale parameters, $\tau_{\phi }$ and $\tau_{\theta }$. We include a separate intercept for each fully connected component of the graph structure embedded in W [56], meaning that the continental U.S. and Hawaii (represented by dummy variables A), respectively, have their own intercepts ($\alpha ={\left[\alpha_{1},\alpha_{2}\right]}^{\prime }$). We also follow Freni-Sterrantino, Ventrucci, and Rue [56] in adjusting the scale of each connected component to render the prior distributions for scale parameters approximately equivalent across any valid spatial connectivity structure [57]. The ICAR model assigned to ϕ is, effectively, two separate models, one for the counties of the continental U.S., and another for Hawaii, each with its own scale parameter ($\tau_{\phi }=[\tau_{\phi_{1}}$, $\tau_{\phi_{2}}$]). To model SA in our covariate [37] (pp. 10–18), [42,58,59], we add its mean spatially-lagged value, WX, as an additional covariate with coefficient γ (recall that W is row-standardized) [38]. Our data and process models for county mortality rates are as follows:

(8) $$\begin{array}{cc} {}[\mathrm{Data}\;\mathrm{model}]:\;X & ∼Gauss(X,S)\\ {}[\mathrm{Process}\;\mathrm{model}]:\;Y & ∼Poiss(\lambda \cdot P)\\ \log(\lambda ) & =A\alpha +\phi +\theta +\gamma WX+\beta X\\ X & ∼MVGauss(1\mu ,{\left(I-\rho W\right)}^{-1}M) \end{array}$$

We mean-center the ICE so that the intercepts α represent the mean log-mortality rates for their respective geographic areas. The remainder of the parameter model is diffuse or weakly informative [60] (p. 19), [61] (p. 55) relative to natural constraints on the data values (e.g., the ICE, and hence its mean μ, is between −1 and 1) or substantive limitations (e.g., the mean log-mortality rate $\alpha_{1}$ must be negative):

(9) $$\begin{array}{cc} \alpha_{1} & ∼Gauss(-5,5)\\ \alpha_{2} & ∼Gauss(0,5)\\ \gamma & ∼Gauss(0,5)\\ \phi & ∼ICAR(\tau_{\phi })\\ \theta & ∼Gauss(0,\tau_{\theta })\\ \mu & ∼Gauss(0,0.5)\\ \tau_{\phi_{1}},\tau_{\phi_{2}},\tau_{\theta },\tau_{X}{]}^{\prime } & ∼Gauss^{+}\left(0,1\right) \end{array}$$

Note that the prior for $\alpha_{1}$ is essentially uniform over the full range of possible values for the mean log-county mortality rate. We also compare results from the full HBM as specified above to a naive model that has the same specification except for the replacement of raw ACS estimates X for the CAR data model of X.

Other valid model specifications are available. The BYM component of the model, in particular, is widely used in the literature, mainly because it tends to be more efficient than using a proper CAR model. Our spatial connectivity matrix was built using an adjacency structure, and was supplemented by manual adjustments for certain low-population areas with missing neighboring observations. A downside of the adjacency method is that it may induce ‘information sharing’ and, potentially, ‘smoothing’ over neighboring observations that are highly dissimilar in terms of demographics [35] (pp. 245–249). For example, it may be undesirable to specify the same degree of connectedness between a majority Native American county and its neighboring, majority White counties, as one might specify between any number of majority White counties, because it conflicts with our knowledge that such populations are not subject to similar sociopolitical conditions. If the purpose of a model is to determine health service provision, for example, then such a choice could have detrimental (and unwarranted) impacts. Our model is primarily for demonstration purposes, and we emphasize that many other modeling purposes require closer attention be paid to such questions.

### 4.3. Results

For each model, we drew 1500 samples from the posterior distributions of parameters for each of 5 independent chains, that after discarding the first 1500 samples of each chain (the burn-in periods). To evaluate MCMC convergence, we use the split $\hat{R}$ diagnostic, which approaches 1 when chains converge on a single distribution; all of our $\hat{R}=1\pm 0.03$ [61]. We require high bulk and tail-area effective sample size (ESS) for mortality rates λ in order to conduct reliable inference on RIIs; both bulk and tail area ESS were >1900 for all λ, which is more than sufficient. We also verified that the residuals from the model contain neither SA nor any indication of non-linearity in the relationship between the ICE and log-mortality rates. The computations were completed using parallel processing and the cmdstanr R package [62] on a Dell XPS 13 laptop computer with Intel Core i7-8565U CPU 1.8 GHz, requiring ≈5.25 h per model.

Table 4 reports a summary of the posterior distributions of select model parameters. The mean county female mortality rate for ages 55–64 is found to be $e^{-4.863}=773$ per 100,000 [770, 776] for the U.S. mainland. Female mortality in Hawaii is estimated to be $e^{-0.162}=0.85$ [0.79, 0.91] times female mortality in the (48) continental states. For males, the corresponding mean county mortality rate is 1259 per 100,000 [1254, 1264], with no difference in Hawaii. For both male and female mortality, the scale of the spatial components of the BYM model, $\tau_{\phi_{1}}$ and $\tau_{\phi_{2}}$, are substantially larger than the spatially unstructured component $\tau_{\theta }$. Thus, net of the ICE, large-scale regional trends account for more variation in county mortality rates than do heterogeneous local characteristics.

Table 5 reports select quantiles of mortality rates for all counties with corresponding RIIs. When ordered by their estimated mortality rates, both male and female mortality have equivalent relative rates: ${\mathrm{RII}}_{5}=1.66$ and ${\mathrm{RII}}_{10}=2.11$. At the extreme, however, the ${\mathrm{RII}}_{100}=\frac{p99}{p1}=3.60$ [3.48, 3.72] for females and 3.68 [3.57, 3.81] for males. Table 6 reports the ICE-Mortality gradient β in terms of the implied RIIs for select quantiles; for female mortality, ${\mathrm{RII}}_{5}=1.35$ [1.33, 1.36], whereas for males ${\mathrm{RII}}_{5}=1.38$ [1.37, 1.40]. These estimates are near the low end of our prior probability distribution for β.

**Figure 12:**
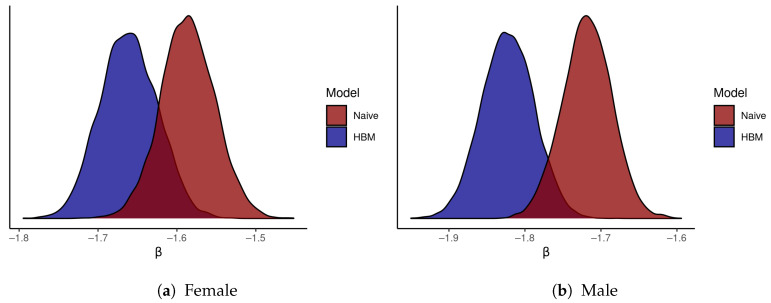
A comparison of the posterior probability density for β, the ICE-mortality gradient, given the full HBM with its density given only a ‘naive’ spatial model that ignores observational uncertainty.

**Figure 13:**
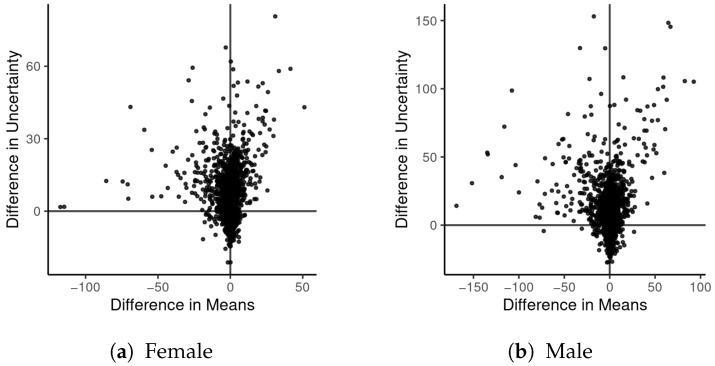
Differences in posterior distributions of mortality rates $\left(\frac{\lambda \cdot P}{100,000}\right)$ for the full HBM and the naive model (difference = full-naive). The mean refers to the mean of the posterior distribution of each rate; uncertainty is measured by the width of the corresponding 95% CIs.

Figure 12 compares the posterior distribution for β under the preferred HBM and under the naive model. For male and female mortality, the distribution is shifted towards zero (attenuated) by the naive model. For the male mortality models, the bulk of the posterior distributions do not overlap each other. Figure 13 shows how uncertainty of the ICE values impacts the probability distributions for the county mortality rates, both in terms of their mean values (estimates) and the width of their 95% CIs (posterior uncertainty). Most of the estimates do not substantively differ, but do have wider CIs. For 41 counties, the absolute difference in male mortality estimates between models is greater than 50 per 100,000, with the maximum absolute difference being 168 per 100,000. The maximum difference in uncertainty for male mortality rates between models is 153 per 100,000, although 69 counties have a difference in uncertainty greater than 50 per 100,000. The largest differences in estimated mortality appear, not surprisingly, in the same counties that have the largest ${\hat{\Delta }}_{i}$ from the ICE data model. Note that many of the same counties that have large ICE SEs also have suppressed female mortality data; therefore, differences between models of female mortality are slightly less than are the differences between male mortality models.

## 5. Conclusions

As access to spatial data products increases, researchers need to be aware of the tradeoffs between data granularity and data quality. This paper offers a methodology for both evaluating data quality and modeling observational uncertainty with spatial survey data, following previously published research on HBMs for spatial data [19,20]. The main contributions of this paper are identifying basic inferential challenges that arise from measurement error with spatial data, and integrating spatial HBMs for survey data into a practical workflow for population health research. Our online supplementary material provides the computer code required to implement the proposed model using the Stan programming language [39].

As researchers aim to take full advantage of new geospatial data products for “precision public health” [63], we caution that data for vulnerable and marginalized populations tends to be the least reliable. Similarly, data quality may impose limitations on our ability to undertake ‘complex’ multivariate analyses. The impacts of observational error on multivariate models are unpredictable; with SA, ignoring such errors may be more treacherous. When a conventional geospatial analysis produces anomalous or implausible results, e.g., that a higher rate of health insurance coverage increases mortality rates in southern Florida [64], researchers and reviewers ought to ask if observational error could be the cause of the findings. Our demonstration analysis shows that ignoring observational uncertainty in a single covariate measured with a fair degree of precision can impact coefficient estimates, model predictions, and posterior uncertainty of estimates. The honest and complete reporting of uncertainty is a critical component of the scientific process and research integrity. Currently accepted practices for analyzing small-area data fall short of this standard, and the widespread adoption of workflows and modeling strategies that incorporate observational uncertainty is called for.

Our analysis of mortality rates benefits from important computational advances of recent years, namely in the application of dynamic Hamiltonian Monte Carlo algorithms to MCMC sampling and Bayesian inference [39,65,66,67]. Nonetheless, computational limitations remain. ACS estimates of population at risk are also subject to sampling error, and it is concerning that researchers employ small-area estimates for highly specific demographic subgroups without considering data quality. Unfortunately, incorporating data models for population at risk estimates into models for count outcomes appears to be a computational bottleneck. The computational limitations of the CAR model also influenced our decision to employ the BYM model specification. Future research may address these challenges. Furthermore, our methodology does not consider the possibility of non-sampling errors in survey estimates. It is possible that errors themselves are correlated due to bias in the sampling design or survey implementation. Modeling potential biases in survey estimates would require additional information.

We recommend that researchers consider data quality to be a core criterion for variable selection, and integral to study design. Analyses of data quality should appear in research protocols, and should be included in the peer review process. The workflow we introduce here is intended to provide a basis for more intensive evaluation and criticism of model-based inferences with small-area data, and to produce models that maintain greater fidelity to researchers’ state of knowledge. When policy and funding decisions are at stake, closer evaluation of priority areas and areas with questionable data quality should be undertaken.

## Figures and Tables

**Table 1:** Degree of SA in select ACS estimates and their respective measures of data reliability (CV for income, SEs for the others).

	U.S. Counties			Milwaukee County Census Tracts		
	Income	Insurance (%)	College Ed. (%)	Income	Insurance (%)	College Ed. (%)
black Estimates	0.80	0.76	0.69	0.84	0.82	0.93
Data reliability	0.56	0.69	0.57	0.46	0.59	0.52

Note: The values reported are estimates of the SA parameter ρ from an intercept-only SAR model, which range from $\frac{1}{\lambda_{min}}$ to 1, where $\lambda_{min}$ is the most negative eigenvalue of matrix *W* (and, $\lambda_{max}=1$).

**Table 2:** Relative magnitude of the median data quality measure (CVs for income, SEs for the others) by ACS variable, geography, and majority racial-ethnic group.

	Income	Insurance (%)	College Ed. (%)
	U.S. Counties		
White	1	1	1
Black	2	1.36	1.17
Hispanic	1.67	1.72	1.11
Native American	1.67	1.85	1.22
	Milwaukee County Census Tracts		
White	1	1	1
Black	1.625	1.5	0.85
Hispanic	1.38	2.39	0.59
Native American	-	-	-

Note: Black, White, and Native American all indicate non-Hispanic identifying populations.

**Table 3:** Summary statistics for select ACS variables, Milwaukee County census tracts, comparing raw ACS data to the posterior distribution of the CAR data model for the same variables.

		Mean	Standard Deviation	SA (ρ)
Log-income	ACS	10.69	0.49	0.88
	Model	10.7 [10.68, 10.71]	0.46 [0.45, 0.48]	0.90 [0.88, 0.91]
Insurance (%)	ACS	91.96	5.67	0.82
	Model	92.81 [92.52, 93.09]	4.59 [4.22, 4.96]	0.89 [0.86, 0.92]
College (%)	ACS	28.31	20.87	0.93
	Model	28.1 [27.69, 28.51]	20.63 [20.23, 21.02]	0.94 [0.93, 0.95]

Note: The model results are summarized here by their posterior means and 95% CIs. The SA parameter ρ is from an intercept-only SAR model; we obtained a posterior distribution for this value by employing the same method used for the mean and standard deviation, i.e., the summary statistic was calculated for each of M=7500 samples drawn from the joint posterior distribution p(X|X,S,I), resulting in a vector of *M* values proportional to the posterior distribution of the summary statistic. Each vector is summarized here by its mean and quantiles.

**Table 4:** Summary of posterior distributions of scalar parameters in the male and female county mortality models.

	Female			Male		
	Mean	2.5%	97.5%	Mean	2.5%	97.5%
$\alpha_{1}$	−4.863	−4.867	−4.859	−4.375	−4.379	−4.371
$\alpha_{2}$	−0.163	−0.236	−0.090	0.005	−0.068	0.077
γ	0.333	0.218	0.449	0.443	0.341	0.549
β	−1.661	−1.720	−1.601	−1.823	−1.879	−1.766
$\tau_{\phi_{1}}$	0.167	0.153	0.180	0.149	0.137	0.163
$\tau_{\phi_{2}}$	0.223	0.058	0.553	0.200	0.028	0.537
$\tau_{\theta }$	0.059	0.047	0.070	0.067	0.056	0.076
μ	−0.001	−0.042	0.038	−0.001	−0.043	0.039
ρ	0.996	0.992	0.999	0.996	0.993	0.999
$\tau_{X}$	0.173	0.169	0.177	0.173	0.169	0.177

**Table 5:** County mortality rates, ages 55–64, by sex and select quantiles and the corresponding relative indices of inequality (posterior means with 95% CIs).

a. Mortality Per 100,000						
	1%	10%	20%	80%	90%	99%
F	391 [382, 400]	529 [523, 536]	602 [596, 607]	1001 [993, 1010]	1116 [1105, 1128]	1408 [1,375, 1,444]
M	627 [612, 641]	865 [855, 874]	980 [972, 989]	1631 [1619, 1644]	1823 [1807, 1840]	2,309 [2261, 2361]
**b. Relative Index of Inequality**						
	$\frac{p80}{p20}$		$\frac{p90}{p10}$		$\frac{p99}{p1}$	
F	1.66 [1.64, 1.68]		2.11 [2.08, 2.14]		3.60 [3.48,3.72]	
M	1.66 [1.64, 1.68]		2.11 [2.08, 2.14]		3.68 [3.57, 3.81]	

**Table 6:** ICE-Mortality gradient summarized as equivalent relative index of inequality for select quantiles.

	$\frac{p20}{p80}$	$\frac{p10}{p90}$	$\frac{p1}{p99}$
F	1.35 [1.33, 1.36]	1.62 [1.59, 1.65]	2.84 [2.72, 2.97]
M	1.38 [1.37, 1.40]	1.70 [1.66, 1.73]	3.14 [3.01, 3.28]

Note: Samples from the posterior distribution of *β* were transformed using *g*(*β*) = *RII* = exp(*dx* × *β*)^−1^. The values reported here are the mean and 95% CI for *g*(*β*).

## Data Availability

The data and computer code presented in this study are available in the supplementary material and an online repository, https://github.com/ConnorDonegan/survey-HBM, accessed on 25 June 2021.

## Abbreviations

The following abbreviations are used in this manuscript:

ACS	American Community Survey
CAR	Conditional autoregressive
CI	Credible interval
CV	Coefficient of variation
ICAR	Intrinsic conditional autoregressive
ICE	Index of Concentration at the Extremes
HBM	Hierarchical Bayesian model
HH	Household
MAD	Median absolute deviation
Markov chain Monte Carlo	MCMC
RII	Relative index of inequality
SA	Spatial autocorrelation
SE	Standard error

## Appendix A. Inference from Uncertain Observation

This appendix summarizes the fundamental logical challenges introduced by observational uncertainty, building upon Polya’s [68] patterns of plausible reasoning. We link this content to the Bayesian theory of ‘probability as extended logic’ [33,68,69,70,71,72,73,74]. We then delineate the frameworks that previous authors have proposed to build Bayesian probability models that incorporate observational uncertainty.

### Appendix A.1. Plausibility

Recall the demonstrative logic of the “modus tollens”:

(A1) $$\begin{array}{c} A\;implies\;B\\ B\;is\;false\\ A\;is\;false, \end{array}$$

where the statements represent, respectively, a premise, an observation or fact, and their logical implication. It states that the falsification of a prediction implied (entailed) by a theory leads to its rejection. More often, we face variants of Polya’s [68] “fundamental inductive pattern,” where the verification of a consequence lends a theory credibility:

(A2) $$\begin{array}{c} A\;implies\;B\\ B\;is\;true\\ A\;is\;more\;credible. \end{array}$$

Because conditions of observation can never be completely controlled (nor, by extension, can they be exactly reproduced) [74], a scientific theory may be compatible with a range of outcomes. Yet, if true, the theory would render certain outcomes more plausible or logically more ‘likely’ than others. Reasoning with such ‘shaded consequences’, to adapt Polya’s [68] terminology, takes elementary forms, such as (cf. [68], pp. 28–37):

(A3) $$\begin{array}{c} A\;implies\;B\;is\;more\;likely\\ B\;is\;true\\ A\;is\;somewhat\;more\;credible. \end{array}$$

The three statements involved in this syllogism correspond to what we commonly refer to as, respectively, the likelihood, the data, and the inference. (This interpretation of likelihood may be found at least as early as Laplace [69]. He described it as our manner of considering, “the variable and unknown causes ...which render uncertain and irregular the march of events” [69] (p. 60). The predominant interpretation of likelihood adopted by the discipline of statistics, and the terminology itself, was introduced by Fisher [75,76]. The term was adopted by Jeffreys [71] (pp. 28–29), but with a Laplacian interpretation. Lipton [77] (pp. 103–120) also explores likelihood as a category of logic, relating Bayesian inference to abductive reasoning.)

The logic of Equation (A3) implies that the observations B correspond directly to the state of reality; that is, our observational process was not obstructed, incomplete, approximate, or displaced (in time or space) from the actual process of interest. With observational uncertainty, our data does not yield ‘B is true,’ but, instead, our data merely renders the proposition B more or less credible. Combining such ‘shaded observations’ with shaded consequences produces ‘twice-shaded’ inductive patterns that take elementary forms, such as:

(A4) $$\begin{array}{c} A\;implies\;B\;is\;more\;likely\\ B\;is\;more\;credible\\ A\;is\;somewhat\;more\;credible. \end{array}$$

This formulation elucidates the inferential challenge of observational uncertainty, and distinguishes it from other sources of uncertainty. In particular, we have differentiated observational uncertainty (’shaded observations’) from likelihood (’shaded consequences’), describing the independent contribution each makes to the uncertainty of our inference. Addressing this challenge requires a valid method for evaluating the weight of evidence given multiple, independent sources of uncertainty.

### Appendix A.2. Probability

As Polya [68] (pp. 109–142) found, patterns of plausible inference may be expressed by algebraic manipulation of the sum and product rules of probability (cf. [33,73,74]). Here, probability is defined to be a logical relationship between two or more considered propositions [70], specifically the rational degree of belief that is afforded to one proposition given the information contained in other true or hypothetical propositions (Following Laplace [69], Jeffreys advanced Bayesian inference as “an extension of logic” [71] (p. 40), [33,68,69,70,72,73,74]. Introductions to this school of thought include [34,78,79,80]. See Greenland [81] for a rival Bayesian paradigm.) A good model, then, is one that faithfully expresses a particular state of knowledge. To enable extensive mathematical expression, we represent impossibility as zero, and certainty as one.

With hypothesis *H*, observation *D*, and background information *I*, we denote the probability of *H* given both *D* and *I* as p(H|D,I). *I* denotes the contextual information without which the problem remains undefined, including that required to specify the probabilities of various outcomes conditional on the truth of *H*, p(D|H,I), as well as any information impinging upon the initial plausibility of *H*, p(H|I) [33,71]. Following Cox’s [73] (pp. 1–4) second axiom of probability, the joint probability of more than one proposition is uniquely defined by the product rule,

(A5) p(H,D|I)=p(D|H,I)p(H|I)=p(H|D,I)p(D|I).

This allows us to evaluate a chain of inferences, as required. We can derive Bayes’ theorem [82] by simple algebraic manipulation of Equation (A5):

(A6) $$p\left(H|D,I\right)=\frac{p(D|H,I)p(H|I)}{p(D|I)}.$$

Bayes’ theorem may be reduced to

(A7) p(H|D,I)∝p(D|H,I)p(H|I),

or, more simply [71]:

(A8) Posterior∝Likelihood×Prior.

Bayes’ theorem states that, after observing data *D* with information *I*, the probability of *H* is proportional to the probability that *D* would arise if *H* were true (the likelihood) times the initial probability of *H* being true (the prior).

### Appendix A.3. HBMs

By successive application of the product rule, one can incorporate ever more information into an inference, to better represent, and update, one’s state of knowledge. As noted in Section 3.1, this causes the joint probability to expand into numerous terms, leaving Equation (A8) wanting for clarity. In its place, we prefer [17,18,20]:

(A9) Posterior∝[Datamodel]×[Processmodel]×[Parametermodel].

Using information on the reliability of observations *D* (the measurement/data model), knowledge of the process of interest D (process model), and the initial plausibility of various states of that process ζ (the exposure/parameter model), one may obtain the relative plausibility of various states of reality, {D,ζ}, given information, {D,I}, by calculating

(A10) p(D,ζ|D,I)∝p(D|D,ζ,I)p(D|ζ,I)p(ζ|I).

Often, we have no direct interest in ζ. Our state of knowledge of D alone is:

(A11) p(D|D,I)∝∫p(D,ζ|D,I)dζ.

For instance, if *D* consists of survey estimates of the poverty rate in all *n* census tracts of a city, then the posterior probability of D is the joint probability distribution of tract-level poverty rates at the time of the survey. Equation (A9) is analogous to Shannon’s [83] concept of the joint entropy of a communication system with noise. Weaver’s introductory comments, in particular, anticipate the fundamental implications of inference with observational error, including variance inflation as well as the value of redundancy in recovering a corrupted message [83] (pp. 18–22). Whereas the rules of grammar and spelling introduce redundancy into written language, SA introduces redundancy into social, health, and environmental variables. The HBM presented in Section 3 exploits this redundancy to improve the quality of, and reduce uncertainty in, the analysis of survey data.

We can expand the model using the product rule again to reason about epidemiological theories. Say a theory *H* posits an ecosocial process linking an exposure, X, to a health outcome, Y. To ease notation, D={Y,X}. The process model must specify the plausibility of various process values, D, conditional on both ζ and the correctness of the theory: p(D|H,ζ,I). With observations D={Y,X}, we would calculate the probability of the theory being true by Bayes’ theorem, Equation (A9), and the rule of total probability, as follows:

(A12) p(H,D,ζ|D,I)∝p(D|D,ζ,I)p(D|H,ζ,I)p(H,ζ|I)

(A13) p(H|D,I)∝∫∫p(H,D,ζ|D,I)dDdζ

Equation (A12) specifies the joint probability of our theory *H* and (descriptive) state of reality {D,ζ}, whereas Equation (A13) integrates over all values of D and ζ to find the marginal probability of *H*. Practically speaking, *H* will consist of one or more parameters in the process model such as a regression coefficient or a risk surface.

To generalize, note that *all* inference implies a data model of sorts and we can denote the certainty of perfect observation as:

(A14) $$p\left(D|I\right)=\left\{\begin{array}{c} 1\;\mathrm{for}\;D=D\\ 0\;\mathrm{for}\;D\neq D. \end{array}\right.$$

Bayesian inference allows us to drop that assertion and jointly consider our uncertainty due to imperfect observation and the logical implications of our observations for our research question, given relevant contextual and background knowledge. Non-Bayesian methods have also been proposed to incorporate observational uncertainty with spatial data, including simulation extrapolation (SIMEX) [10,84] and empirical Bayesian methods [20] (pp. 23–24). However, neither can fully and consistently propagate uncertainty of all parameters into the final results. The automatic propagation of uncertainty from intermediate inferences is a critical advantage of HBMs.

## Appendix B. Between-County Inequality: Prior Knowledge

Table A1 presents the findings from each of the eight studies that were found to provide estimates of the magnitude of relative cross-county inequality of county mortality rates. As indicated by the ‘Comparison’ column of Table A1, the authors have grouped counties into bins, such that the bottom quintile contains all counties below the 20th percentile. Note that this contrasts with the method we employ, which is to compare the fitted values of specific percentiles. One advantage of this approach is that we can evaluate $\frac{p10}{p90}$ separately from other cut-points, e.g., $\frac{p1}{p99}$, instead of averaging over them. Despite heterogeneity of methods and time period, the published findings on relative inequality fall within a fairly narrow range of RRs (as noted in Section 4.1). The most extreme finding [51] was a RR of 2.7; however, the methodology differs considerably from ours. The authors grouped counties using a clustering algorithm that considered county mortality rates, their trajectories over time, and other terms.

In addition to published findings on county mortality rates, our prior model for β also reflects the large body of historical and contemporary literature that finds mortality to be inversely associated with social class and wealth [1,4,5,85,86,87,88], which precludes us from assigning any substantive amount of prior probability to positive values of β. We do not assign appreciable prior probability density to arbitrarily large *negative* values of β either. We aim to assign appreciable prior probability to all plausible values of β, given the peer-reviewed literature on the topic and allowing for some additional uncertainty due to the small number of directly comparable studies. Note that the bulk of our prior probabilty model for β covers the entirety of the posterior probability density. Thus, the prior for β did not pull the estimates of β in one direction or another relative to the process models (i.e., the likelihood). With over 2800 observations, the prior for β is “dominated” by the likelihood (see [60], pp. 18–19). Nonetheless, encoding substantive prior information into probablity distributions is good practice because it may yield additional insight into final results, it may help us become better acquainted with the formal and substantive meanings of our model parameters, and it is the only way to ensure that a parameter model is not unreasonable.

**Table A1:** Summary of select published findings on inequality in all-cause county mortality.

Source	Outcome	Period	Comparison	Findings
[47]	Age-adjusted mortality, 0–19 yo.	1968–1992	Bottom v. top quintile by deprivation index	y. 1992 RR = 1.52; RR range: 1.45–1.65
[48]	Age-standardized mortality, 0–65 yo.	1960–2002	Bottom v. top quintile of median HH income	RR = 1.6 [1.6, 1.7]
[45]	Age- and sex-adjusted mortality	1988–1992	Lowest inequality, highest income v. highest inequality, lowest income; by quartile of each measure	RR = 1.22
[50]	Mortality, 15–24 yo.	1999–2007	Bottom v. top decile by deprivation index	MaleRR=1.90[1.87,1.94]; Female RR = 1.62 [1.56, 1.67]
[51]	Age-adjusted mortality	1999–2013	Highest v. lowest of 8 county classes, grouped by mortality	RR increased each year from 2.1 (1999) to 2.7 (2013)
[49]	Age-standardized mortality, 25–64 yo.	2000–2003	Quintiles of education (% bachelors degree+) and median HH income	Education RR = 1.64; Income RR = 1.78
[46]	Age-adjusted mortality, 0–75 yo.	2002–2006	Bottom v. top quartile of median HH income	RR = 1.41
[52]	Mortality, 45–64 yo.	2005–2009	Metropolitan areas with <5% poverty vs. non-metro. areas with >20% poverty	RR = 2.22 [2.20, 2.24]

Note: All reported uncertainty intervals are 95% confidence intervals. Findings based on the same mortality data as the present study are excluded from this summary to avoid allowing our data to inform our prior probability model.
